# Dialectical behavioral therapy skills training in assisted living: transdiagnostic effects on goal attainment, self-efficacy, and symptom severity

**DOI:** 10.3389/fpsyt.2025.1669524

**Published:** 2025-10-07

**Authors:** Philipp Koziol, Carina Florin, Kathleen Heft, Robert Fellinger, Thomas Probst

**Affiliations:** ^1^ Division of Psychotherapy, Department of Psychology, University of Salzburg, Salzburg, Austria; ^2^ Pro Mente Salzburg, Salzburg, Austria

**Keywords:** dialectical behavior therapy, skills training, assisted living, psychological distress, self-efficacy, goal attainment, transdiagnostic intervention

## Abstract

**Background:**

Dialectical Behavior Therapy Skills Training (DBT-ST) is an evidence-based intervention targeting emotion regulation, self-efficacy, and psychological well-being. While widely applied in clinical settings, its effectiveness and feasibility in long-term residential care remain underexplored. This study hypothesized that an 8-week DBT-ST program would increase goal attainment and self-efficacy, and decrease psychological distress among assisted living residents, with further improvements expected at a two-week follow-up.

**Method:**

A total of 52 residents from four assisted living facilities operated by Pro Mente Salzburg, Austria, participated in an 8-week DBT-ST program. The intervention was delivered by the facilities’ psychologists and by staff supervised by psychologists in weekly group sessions. Self-reports were used to assess goal attainment (ranging from 0% to 100% in 10% intervals), self-efficacy (Self-Efficacy Scale – Short Form; ASKU), and psychological distress (Kessler Psychological Distress Scale; K10).

**Results:**

Goal attainment (p = .040, d = 0.28) and psychological distress (p =.034, d = 0.26) showed initial improvements from baseline to post-intervention, but these effects did not withstand Bonferroni correction. No significant changes were observed between post-intervention and follow-up.

**Conclusions:**

While nominal improvements were observed, these did not remain significant after correction for multiple testing. The study nonetheless offers preliminary evidence on the feasibility and the challenges of DBT-based interventions in assisted living settings and highlights the need for future research on their context-sensitive adaptations.

## 1 Introduction

Dialectical Behavior Therapy (DBT) was developed in the late 1980s by Marsha Linehan addressing the pervasive challenges of borderline personality disorder (BPD), self-harm, and suicidal behavior. It combines traditional cognitive-behavioral techniques with mindfulness and acceptance-based strategies. This new theoretical and practical framework contributed to the emergence and consolidation of third wave cognitive behavioral therapy approaches. The pathology of BPD is characterized by profound instability both within and between emotional states leading to volatile, maladaptive, and potentially outright dangerous behavior. Thus, DBT is conceptualized to emphasize the dialectical principle of integrating opposites, i.e., finding balance between acceptance of emotional experiences and the need for change.

A fundamental component and principal method of delivery is DBT Skills Training (DBT-ST). It is typically delivered in a group format and focuses on imparting practical skills to replace maladaptive behaviors. Clients are introduced to a broad repertoire of skills and practice them in a non-stressful and supportive environment. They proceed to select and streamline those that are relevant to their individual challenges with their DBT therapist. Teaching skills in a group setting offers several crucial advantages. Participants can connect with others facing similar challenges, nurturing a sense of cohesion and mutual support. It provides them with valuable feedback and, perhaps more importantly, enhances self-efficacy expectations through vicarious experiences in the group. These processes can support clients in refining their own coping strategies and gain better insight into their emotional experiences, thoughts, and behaviors.

DBT-ST is centered around four modules (mindfulness, distress tolerance, emotion-regulation, interpersonal relationships) that, in concordance with the ethos of DBT, convey both “acceptance skills” and “change skills” (1). Mindfulness is unquestionably at the center of DBT and was derived from western and eastern contemplative practices, most notably from Zen-Buddhism. One cannot be expected to enact change or cope with emotional experiences if their current state is not sufficiently perceived and experienced in the moment. For instance, a client will most likely not be able to regulate emotional outbursts, if the underlying emotions and their triggers are not fully perceived, explored and understood. Similarly, someone feeling intense stress might not be able to employ the correct coping strategy, without understanding their physical and psychological sensations. Mindfulness teaches clients to exist ‘in the moment’ and accept ‘what is.’ It forms the basis of subsequent modules and is practiced in every session. Distress tolerance teaches clients a set of skills that are aimed at surviving crises without resorting to maladaptive behavior, such as using drugs, engaging in high-risk behavior or self-harm. Many of these skills focus on alleviating suffering by stimulating the practitioner’s nervous system. This can occur through temperature change, such as holding one’s face in cold water, eating a chili or biting into a lemon. Other skills aim to foster acceptance of painful circumstances that cannot be changed, thereby increasing one’s perceived freedom. The emotion-regulation module emphasizes the evolutionary adaptive value of emotions and introduces a framework for understanding and managing emotions. The fourth module concerns interpersonal relationships. Maladaptive emotional reactions harm relationships or inhibit interactions altogether, leading to avoidance or non-interaction. Thus, this module targets conflict management and relationship skills, aiming to reinforce one’s environment rather than only inner change. Indubitably, this relationship can be seen as bi-directional, as change in our perceived environment affects change in perceptions of and within ourselves and our behavior. This principle is rooted in the works by Kurt Lewin, Albert Bandura, and others (cf. 2). In the German manual for DBT-ST by Bohus and Wolf-Arehult (3) a fifth module was added, labelled “self-esteem”. This serves to sensitize clients to dysfunctional assumptions about themselves and their consequences, as well as foster a positive perception and acceptance of themselves.

Empirical research supports the efficacy of DBT both as an integrated and as a skills-only program. Thus, it was subsequently adapted to the specific needs of pathologies, in particular comorbid PTSD, eating disorders, and substance abuse disorders (1, 4, 5). Those disorders, combined with BPD, represent the largest and most prominent areas of research concerning the efficacy of DBT and DBT-ST by a very substantial margin. In particular, DBT-Skills have been shown to have a significant impact on many of the symptoms of the aforementioned disorders and have been identified as a principal mechanism of change within DBT (5). In similar research, Soler et al. (6) compared DBT-ST to standard group therapy and found reduced depression, anxiety, and psychotic symptoms in people diagnosed with BPD, showing significant difference to the control group. Reductions of other BPD-symptoms like dysfunctional anger, emptiness, and affect instability were observed in the DBT-ST group. Further research shows similar effects of DBT-ST on BPD symptoms (7–9) both in inpatient and outpatient settings. Interestingly, since typically an overwhelming proportion of study participants in BPD settings are female, Birt et al. (10) found significant decreases in emotion dysregulation among men and thus similar emotion regulation capacity compared to an all-female control group.

Beyond emotional unstable personality disorders, DBT-ST has been shown to successfully reduce emotion dysregulation among individuals suffering from alcohol addiction, resulting in a 73% abstinence rate (11). The training program also benefits patients with behavioral addictions (e.g., gambling, sex, shopping), not just those with substance use disorders. Reported effect sizes reach medium levels (12).

Several studies examined the effect of DBT-ST on various primary and secondary outcomes such as binge eating, purging, emotional dysregulation, depressive, and anxiety symptoms. Safer et al. (13) administered DBT-ST to women diagnosed with bulimia nervosa and found significant changes for the Emotional Eating Scale (EES) subscales such as anger, anxiety, depression, and the negative affect subscale of the Positive and Negative Affect Schedule (PANAS) with moderate to large effect sizes. This led them to theorize, that the treatment mechanism lies within a reduction of vulnerability to negative emotions associated with binging and purging, rather than directly targeting areas such as self-esteem and overall impulsivity. However, Telch et al. (14) proposed a different mechanism in their study concerning binge eating disorder. While they found significant effects for binge days and episodes with 89% of participants reaching abstinence (i.e. no binge eating episodes in the past four weeks), they did not find statistical significance regarding anxiety and depression. They surmised that treatment may reduce the urge or impulse to eat when experiencing negative emotions, rather than working directly on the affect. Both teams of authors rejected a direct effect on affective elements and postulate treatment mechanisms through secondary components. This seems consistent with the overall ethos of DBT-ST: participants are being taught skills to better regulate their responses to emotional states, rather than directly altering the emotions themselves.

Several other studies were able to show significant effects on depressive and anxiety symptoms in eating and borderline disorder patients post-DBT-ST (6, 9, 12, 15, 16). Hill et al. (16) adapted DBT-ST for binge eating with purging and found reduced eating disorder symptoms and improved emotional awareness, with half of all participants no longer meeting criteria for bulimia nervosa and a quarter reaching full abstinence. While not a clinical sample, Flynn et al. (17) administered a DBT-ST in Irish post-primary schools and found significant changes in social stress, anxiety, depression, sense of inadequacy, self-esteem, self-reliance, as well as internalizing problems. This alludes to the possibility of DBT-ST having a tremendously beneficial effect if employed proactively during adolescence.

As shown, DBT-ST is being used in a variety of clinical populations with different diagnoses. However, studies that examine the transdiagnostic effect of this program are still scarce. Of the studies that are available, most assume the common transdiagnostic factor of emotion dysregulation, which DBT-ST is designed to target. Neacsiu et al. (18) tested DBT-ST against an activities-based support group (ASG) in participants with at least one current depressive or anxiety disorder. For the ASG, therapists were instructed to nurture a sense of group affiliation, maintain positive relationship, offer empathy and psychoeducational lessons. DBT-skills and cognitive behavioral strategies were prohibited. The study found significant reduction in symptoms for DBT-ST; however, those did not remain stable and matched the ASGs symptom levels at follow-up six months later. The authors theorized that once motivational support and guidance ceases, effects in DBT-ST start to diminish. Durpoix et al. (19) offered DBT-ST to a mixed group of patients with borderline personality disorder, bipolar disorder, and Attention-Deficit/Hyperactivity Disorder (ADHD). According to a self-assessment one year after treatment, 73% of participants judged DBT-ST to be important or very important and 64% said that they were using skills often or very often. Unfortunately, no further data concerning symptoms is available. Furthermore, Vijayapriya and Tamarana (20) conducted a review of twelve studies employing DBT to investigate its transdiagnostic effect in strengthening cognitive functions. Prevalent conditions were emotional dysregulation, borderline personality disorder, bipolar disorder, ADHD, and multiple sclerosis. According to the review’s findings, DBT has the potential to enhance cognitive functions such as attention, memory, fluency, response inhibition, planning, set shifting, tolerance for delayed rewards, and time perception.

Despite this broad evidence base across inpatient, outpatient, and community populations, the use of DBT-ST in assisted living facilities has received little empirical attention. Residents in such settings often present with severe, chronic psychiatric conditions (e.g., schizophrenia spectrum disorders), frequently accompanied by cognitive impairments such as reduced insight, attention, and memory difficulties. Many reside in long-term or lifelong care contexts that require continuous psychosocial support, distinguishing them from short-term inpatient or outpatient populations. Therapeutic approaches must therefore be integrated into daily routines and adapted to both cognitive and motivational limitations. In addition, implementation relies heavily on the involvement of staff, who play a central role in supporting skill practice and reinforcing use outside of training sessions. Investigating DBT-ST in this context is essential to evaluate its feasibility, adaptability, and potential benefits for individuals who are unlikely to access or sustain participation in more traditional treatment formats. Given the scarcity of research in this area, the current study aims to examine the effects of an abbreviated DBT-ST program in a transdiagnostic sample in assisted living facilities. The goal was to provide valuable insight into its feasibility and potential benefits in complex care settings. The decision to conduct this study in an environment that houses residents suffering from severe psychological disorders that massively interfere with everyday life was informed by the observation that such populations are rarely included in treatment effectiveness research. This study seeks to address this gap and to encourage further research and tailored intervention development for both assisted living residents and transdiagnostic applications more broadly. Three outcome domains were investigated for this purpose and will be discussed in the following sections: goal attainment, self-efficacy, and psychological distress (as an index of symptom severity).

### 1.1 Goal attainment

Many studies that investigated the effects of DBT interventions – and many therapists in the field – make use of diary cards (e.g., 8). Patients can document and track their skill use across days of the week. In fact, patients fill out these measurement tools themselves, as this helps them to visualize their progress and can be a strong motivational factor in therapy. Often, a Goal Attainment Scale (GAS) is additionally utilized to monitor their progress. Patients initially set a target goal and track their progress using percentage ratings for each day.

### 1.2 Self-efficacy

Albert Bandura postulated that belief in oneself plays a key role in regulating behavior as early as 1971 (21). He coined the term *self-efficacy* in subsequent works as a principal component of his Social Learning Theory (22–25) to explain how humans can influence their behavior and, subsequently, their goals. It “refers to beliefs in one’s capabilities to organize and execute the courses of action required to produce given attainments” (25). In other words, it is the belief that one can successfully execute a given behavior required to effect a desired outcome. Bandura is exceptionally specific in his works and distinguishes between the concepts of efficacy and outcome expectations. If a person lacks belief in their ability to perform a behavior (self-efficacy expectation), knowing that it could lead to a desired outcome (outcome expectation) will not influence their actions (e.g., 25).

Bandura (24) discussed self-efficacy in the context of dysfunctional behavior (or apathetic non-behavior), particularly in connection with anxiety. He argued that as self-efficacy increases, anxiety arousal declines and people experience themselves as more capable of managing environmental threats. Hence, distress results from a maladaptation between one’s perceived self-inefficacy and the threat level presented by one’s environment.

Furthermore, Bandura (23) elegantly drew a connection to Seligman’s Theory of Learned Helplessness. According to Seligman (26), individuals become inactive and depressed when they find that their actions cannot influence their environment – that is, what happens to them. He later reformulated his theory, arguing that the cause of those detrimental effects does not stem from the belief that one’s actions will have no effect, but rather from the belief that one cannot perform the required actions (27). One can surmise that individuals are prone to becoming inactive and depressed when they have low self-efficacy. Conversely, their activity is more likely to increase when they have high self-efficacy. Indeed, according to Bandura (21), self-efficacy influences an individual’s choices, the effort they put into chosen tasks, and their persistence in the face of challenges, such that higher self-efficacy leads to the pursuit of more challenging goals and greater commitment to achieving them.

Bandura identified several sources of self-efficacy, the most valuable being one’s own performance or mastery experiences (22). Accordingly, the relationship between self-efficacy and goal attainment can be described as bi-directional: individuals with high self-efficacy seek out incrementally more challenging goals, the achievement of which then, in turn, generates more self-efficacy through mastery experience. This is the principal mechanism of DBT-skills: trainees learn to employ various skills and thereby generate more self-efficacy through mastery experiences. Bandura (22) identifies vicarious experiences as the second most important source of self-efficacy. Observing others succeed and receiving encouragement from fellow participants and skill trainers increases one’s own belief in their own ability to execute the same behavior and accomplish the same task. To facilitate participants’ learning through shared mastery experiences and mutual support, DBT-ST is typically employed in a group setting.

According to various studies cited in Bandura (23), self-efficacy predicts the degree of change in diverse types of social behavior, phobic dysfunctions, stress reactions, physiological arousal, stamina, self-regulation of addictive behavior, achievement strivings, career choice, and development. These effects have been further tested in a myriad of studies to date. Recent research has found it to have beneficial effects on overall mental health (28) and to reduce negative affect associated with depression and anxiety (29–31). Self-efficacy predicts symptom reduction in patients with panic disorder, generalized anxiety disorder, social anxiety disorder, and PTSD (32) and reduces symptom severity in patients with social anxiety disorder (33). It correlates negatively with emotion dysregulation and borderline symptoms (34), predicts earlier relapse in addiction patients (35) and is associated with ADHD symptoms (36). Studies have also demonstrated positive correlations between self-efficacy and social functioning in patients diagnosed with schizophrenia (37, 38). This will be of particular interest in the current study, as the majority of participants are diagnosed with this disorder.

DBT-skills, specifically, have been shown to boost self-efficacy in patients with borderline personality disorder (7) as well as substance abstinence self-efficacy (15, 39, 40). In a population of children diagnosed with ADHD, the use of a conversational agent in the form of a chatbot – which can be interpreted as a skill – led to increased self-efficacy and reduced ADHD symptoms (41).

### 1.3 Psychological distress

Psychological distress has been a topic of vivid discussions among researchers over the past decades. Drapeau et al. (42) defined psychological distress “as a state of emotional suffering characterized by symptoms of depression (e.g., lost interest; sadness; hopelessness) and anxiety (e.g., restlessness; feeling tense)” that is present in most psychological disorders. In their definition, they refer to Mirowsky and Ross (43), who discussed the measures of mental health in sociology and plead for the use of indexes instead of diagnoses, since diagnoses fail to paint an exhaustive picture of human suffering. As such, they are two of a seemingly growing number of researchers, calling for a shift from dichotomous to continuous outcome variables. In fact, the use of psychological distress in sociology, in particular, has been heavily criticized by Horwitz (44). He lamented the imprecise conceptual frameworks researchers in the field of sociology have been operating under. He argued that their studies often conflate psychological distress with mental disorders, failing to recognize that distress can occur in healthy individuals without leading to dysfunction. This blurring of categories can lead to treating normal emotional reactions as diseases and thus overstating the prevalence of mental disorders in the general population.

Horwitz characterized psychological distress as a transient phenomenon that corresponds to a natural emotional reaction to a stressor. He emphasized this point by citing several studies that seem to show high fluctuations of depressive symptoms among adolescents over intervals as short as one month. He depicted these fluctuations as a relatively brief sorrow following an aversive event such as failing a test, losing in a sports competition or ending a romantic relationship. According to Horwitz, distress should diminish when the aversive stressor is no longer present or when the individual adapts to it. Thus, he conceptualized psychological distress as distinct from psychological dysfunction and from the term and diagnosis of a mental disorder. In stark contrast to Horwitz’s views, Wheaton (45) disputed the transient nature of psychological distress in a comment on his publication. He, in turn, cited seven of his own longitudinal studies that demonstrate distress to be moderately stable over the course of 1 to 10 years. He described psychological distress as an integral part of depression and anxiety and explicitly not “normal”, as Horwitz suggests. This last point was taken up by Drapeau et al. (42) who noted that, although negative affective states such as feeling sad, depressed or anxious are viewed as universal, there is a distinct cultural variation of how those states are expressed. Furthermore, they noted that Wheaton’s studies could clarify the role of personality in the stability of psychological distress. They argued that neuroticism has been demonstrated to be linked to psychological distress and may partly even account for its chronicity.

To resolve the disparity between the two different conceptualizations, distress may be transient until it is not. Distress could appear in a manner as Horwitz argued and may stabilize as a consequence of persistent stressors and the individual’s inability to adapt, thus becoming part of the mental disorder. This would entail that psychological distress is not separable from the concept of disorder as Horwitz advocated. In fact, Phillips (46) discussed the operationalization of distress and recommended the use of distress as a dimensional appraisal of functional impairment (i.e., symptom severity) for all disorders.

In the research on DBT-skills and their effect on psychological distress, only one study by Wieczorek et al. (47) measured psychological distress in this manner, using the Kessler 10 scale (K10; 48). The study showed significant reductions in psychological distress at post-treatment measurement for individuals experiencing pervasive emotion dysregulation symptoms, deliberate self-harm or suicidal behaviors. Following both Wheaton (45) and Phillips (46) approaches, and the example of Wieczorek et al. (47), measuring psychological distress as an index for symptom severity is deemed appropriate for the current study.

### 1.4 Hypotheses

Based on the theoretical framework outlined above, the present study aims to investigate the effects of an adapted, 8-week Dialectical Behavior Therapy Skills Training (DBT-ST) program on symptom severity, self-efficacy, and goal attainment in a population of assisted living residents. The hypotheses are as follows:

H1a: Psychological distress, as an indicator of symptom severity, will be significantly reduced at the post-intervention time point (after 8 weeks) compared to baseline (pre-intervention) levels.
H1b: Psychological distress will show a further significant reduction at follow-up (2 weeks after the post-intervention time point) compared to post-intervention levels.
H2a: Self-efficacy will significantly increase at the post-intervention time point compared to baseline levels.
H2b: Self-efficacy will significantly increase at follow-up compared to post-intervention levels.
H3a: Goal attainment will be significantly higher at the post-intervention time point compared to baseline levels.
H3b: Goal attainment will be significantly higher at follow-up compared to post-intervention levels.

## 2 Materials and methods

The study was approved by the ethics committee of the University of Salzburg, Austria (GZ 57/2024). All participants provided informed consent to take part in the present study.

### 2.1 Study design

This study was conducted as a multicenter (four assisted living facilities), longitudinal pilot feasibility trial. It employed a pre-post-follow-up design without a control group to test effects of a face-to-face intervention (DBT-ST). The absence of a control condition represents a major limitation, as casual inferences cannot be drawn; however, the design was deemed appropriate to generate preliminary feasibility data in this under-researched setting. The intervention phase comprised 8 weeks, the follow-up period two weeks after the intervention. The skills trainers were introduced to the DBT-ST protocol through a structured preparatory briefing to ensure consistency across facilities and to familiarize them with the specific structure and content of the intervention. All necessary materials were provided to the skill trainers at this point. One week later, the lead researcher (first author) introduced residents to the general concept of skills training, defining skills and their use as coping strategies. This introduction aimed to both inform and motivate residents to participate. Subsequently, participants were fully informed about the study procedures, inclusion and exclusion criteria, measured outcomes, and data security. Skills trainers commenced the intervention program the following week, conducting one 1-hour session per week over the course of eight weeks.

### 2.2 Assisted living facilities

Tauernhof, Südhof, and Aktive Großfamilie are long-term projects, meaning that residents typically spend the remainder of their lives in these facilities. All four facilities (Tauernhof, Südhof, Heimo-Gastager-Haus, and Aktive Großfamilie) are operated by Pro Mente Salzburg, a nonprofit organization providing psychological and social rehabilitation. Each resident is provided with a single bedroom and a small bathroom, while kitchens and various common rooms are shared among all residents. Support includes assistance with organizing recreational activities, managing daily life, (re)learning social skills and interactions, communicating with departments and agencies, setting personal goals, and general 24/7 care, including on-call night shifts. The Heimo-Gastager-Haus is a transitional living facility. The maximum length of stay is 36 months and is intended for individuals who have experienced prolonged clinical stays and were forced to interrupt their occupation or education due to mental health conditions. Residents are supported by psychologists, psychotherapists, consultation-liaison psychiatrists, and social workers to facilitate the resumption of occupational or educational activities. Greater emphasis is placed on readiness for change and active cooperation, and residents are expected to largely care for themselves and manage their own medication schedules.

In each facility, DBT-ST sessions were held in a common room designated for group meetings and therapy sessions, equipped with desks, chairs, and flipcharts. The room was well lit, and external noise did not interfere with the sessions.

### 2.3 Patient sample

Inclusion criteria were to be at least 18 years old and diagnosed with at least one psychiatric ICD-10 diagnosis. Exclusion criteria included acute psychosis, acute risk of harm to self and/or others, insufficient language comprehension due to limited proficiency or neurological disorders (e.g., aphasia following a stroke or dementia), and lack of decision-making capacity if such capacity could not be restored. As the primary caregivers in the assisted living context, the facility staff were instructed to identify and exclude participants meeting the exclusion criteria from the weekly intervention program and to report these exclusions to the study’s lead researcher (first author).

Since the present study was conducted within four assisted living facility (see above), the sample can be classified as a convenience sample. After obtaining approval to conduct the study from the deputy CEO of Pro Mente Salzburg and after approval from the ethics committee, managers of each facility were contacted and provided with information about the study procedures and materials. Based on the inclusion and exclusion criteria, they selected appropriate residents to participate in the intervention program. Participants did not receive monetary compensation for their involvement in the study. Instead, they were sincerely thanked for their participation and invited to a social gathering, during which a meal was prepared and shared together as a token of appreciation.

### 2.4 Skill trainer sample

All skill trainers were drawn from the existing staff of the respective assisted living facilities, ensuring familiarity between participants and their trainers. This approach was considered more practical and prudent than introducing external trainers, particularly given the participants’ motivational states, potential difficulties engaging with the material, and the possibility of psychological crises requiring immediate attention.

The pool of skills trainers comprised of post-graduate psychologists, clinical and health psychologists, psychotherapists and social educational workers. Those without advanced training in psychology were supervised by trainers who possessed such qualifications. This supervision structure aimed to maintain integrity and consistency of the intervention across facilities. One was a trainer who was fully certified as a DBT skills trainer, while two others were undergoing training to obtain this qualification.

### 2.5 Intervention

The DBT skills training program employed in the present study was implemented into the weekly schedule of all assisted living facilities and replaced a weekly house group session, ensuring the availability of all residents and minimal disruption to daily life.

The intervention was an adapted version of the interactive skills training for patients with borderline personality disorder developed by Bohus and Wolf-Arehult (3). The manual was shortened to accommodate the eight-week duration, and to suit participants’ cognitive capabilities, considering their severe mental disorders. Content was simplified where necessary, and exercises were selected or modified to ensure accessibility and relevance for a clinical population living in assisted settings.

Formal adherence ratings were not conducted. Instead, supervision was provided through regular team meetings, where trainers in supervision could discuss procedures and difficulties and receive guidance to support consistent delivery.

#### 2.5.1 DBT-Skills-training

The skills training manual used in this study is a condensed, 8-week group intervention adaptation of the *Interaktives Skillstraining für Borderline-Patienten* by Bohus and Wolf-Arehult (3), tailored for residents in assisted living settings. The skills training retained the structure of the original version (3), organizing the intervention into five modules: mindfulness, stress tolerance, emotion regulation, interpersonal skills, and self-esteem. Due to their centrality in DBT, as emphasized by the authors, and their impact on the outcome measures in the studies cited in the introduction of this paper, two of the weekly sessions were allocated to each of the mindfulness, stress tolerance, and emotion regulation modules. One of the weekly sessions dealt with the interpersonal skills module and another of the weekly sessions with the self-esteem module. Mindfulness is considered a fundamental prerequisite for progressing through the remaining modules. The authors of the original manual argue that one cannot adequately begin to manage stress or overwhelming emotions without first mastering mindfulness skills, which are necessary to detect internal physical and psychological changes. To reinforce mastery and encourage continuous mindfulness practice, five minutes at the start of each weekly session are typically reserved for a short mindfulness exercise. The adapted manual used in the present study followed this recommendation. Each session was then structured into two phases:

1. Phase one involved the presentation of new theoretical content using visual aids such as mind maps, tables, graphs, and flipcharts.
2. Phase two consisted of practical skill exercises. Participants were encouraged to review the material, deepen their understanding, and practice the skills between sessions. In addition, trainers were advised to facilitate discussion and reflection, allowing participants to share experiences, ask questions, and connect the skills to their own daily lives. This dialogical component was included to increase participant engagement, foster group cohesion, and promote internalization of the material beyond the training context.

A description of the abbreviated manual is provided below.

Introduction: Orientation and Preparation.

Before the first skills module, participants attend an orientation session. It introduces the purpose and structure of the DBT Skill Training, defining key concepts such as skills, distress curves, dimensions of arousal, sensory channels, and early warning signs.

Module 1: Mindfulness (Weeks 1 & 2).

Participants are introduced to the fundamental DBT mindfulness skills, including the *What* skills (Observe, Describe, Participate) and the *How* skills (Non-judgmentally, Focused, Effectively). Theoretical explanations are paired with practical exercises and reflection tasks to enhance present-moment awareness.

Module 2: Distress Tolerance (Weeks 3 & 4).

This module focuses on acute crisis management. Participants learn short-term strategies to survive emotional crises without making things worse. Skills include grounding through the five senses, breathing techniques, distraction strategies, and guided attention. The “emergency toolkit” exercise consolidates these practices for daily use.

Module 3: Emotion Regulation (Weeks 5 & 6).

Emotion-focused sessions teach participants how emotions function, how they are influenced by thoughts, behaviors, and physical sensations, and how to modulate them effectively. Skills include opposite action, emotional surfing, and building positive emotional experiences. Emphasis is placed on awareness and constructive responses to emotional triggers.

Module 4: Interpersonal Effectiveness (Week 7).

This module addresses core skills for improving interpersonal relationships. Topics include balancing self-respect, relationship goals, and objective effectiveness. Exercises are designed to train active listening, validation, and assertiveness using structured dialogue and roleplay.

Module 5: Self-Esteem (Week 8).

The final module focuses on self-care and self-respect. Exercises include the “InSEL skill”, a German acronym promoting self-compassion and responsibility, as well as a skill to balance frustration. These encourage reflection and the planning of positive experiences to buffer against stressors.

Throughout the program, the manual provides color-coded guidance for skill trainers: green elements for flipchart-based psychoeducation and red elements for worksheets to be distributed in individual participant folders.

#### 2.5.2 Materials

Skill trainers were provided with the adapted manual, questionnaires for ten weeks of data collection, and a box containing basic skill items. The skill items included a stress ball, a spiky massage ball, a ball of plasticine, a bag of cherry stones, a packet of intense lavender ampules, and several acupressure metal rings. The manual provided guidance on conducting the weekly skill training sessions and was color-coded to distinguish between content for theoretical input (to be displayed on flipcharts) and worksheets for participants to complete. Consistent with the original manual by Bohus and Wolf-Arehult (3), the training sessions were designed to be conducted in a pleasant, workshop-style setting with desks, writing utensils, and refreshments to foster a supportive learning environment. This approach aimed to accommodate participants’ cognitive needs and reduce potential resistance by creating a comfortable and low-pressure atmosphere.

### 2.6 Measures

Paper-pencil questionnaires were administered to the patients to assess the following variables by self-report.

#### 2.6.1 Demographics

Participants were asked to provide demographic information, including their age, sex, and whether they were engaged in active psychotherapy at the time of data collection. Diagnostic codes were completed by the skills trainers and were subsequently categorized using their three-character ICD-10 codes.

#### 2.6.2 Goal attainment

Participants defined and wrote down a personal goal that was relatively short-term, measurable, concrete, and something they wished to attain or make progress toward by the end of the intervention. They rated their goal attainment as a percentage, ranging from 0% to 100% in 10% intervals. Goal attainment was assessed at baseline, each session, post-intervention, and follow-up. In the analyses and tables, results are reported as percentages for clarity.

#### 2.6.3 Self-efficacy

Self-efficacy was measured using the Allgemeine Selbstwirksamkeit Kurzskala (Self-Efficacy Scale – Short Form; ASKU; 49). The ASKU economically assesses competence expectancy in coping with daily difficulties. Internal consistency ranges between McDonald’s ω = .81 to ω = .86, indicating good reliability. According to Beierlein et al. (49), the scale’s single factor structure is supported by factor analyses. Convergent and discriminant validity are evidenced by a strong correlation with an alternative measure of general self-efficacy by Schwarzer and Jerusalem (50). The ASKU has been successfully applied in diverse populations, including clinical and non-clinical groups, which supports its suitability for use in the present sample.

The scale consists of three statements: “I can rely on my own abilities in difficult situations,” “I am able to solve most problems on my own,” and “I can usually solve even challenging and complex tasks well.” Participants were asked to assess the extent to which each statement applied to them personally in the past seven days on a 5-point scale, with the following options: “doesn’t apply at all” (1), “applies a bit” (2), “applies somewhat” (3), “applies mostly” (4), and “applies completely” (5). Responses were averaged to yield a total score from 1 to 5. Self-efficacy was assessed at baseline, each session, post-intervention, and follow-up.

#### 2.6.4 Psychological distress

Psychological Distress, as an indicator of symptom severity, was measured using the Kessler Psychological Distress Scale (K10; 48). The K10 was originally developed as an epidemiological tool using Item Response Theory (IRT). A validation study of the German version by Giesinger et al. (51) employed Receiver Operating Characteristic (ROC) curve analysis, revealing excellent detection of mental disorders (area under the curve, AUC = 0.88), supporting the scale’s validity. The instrument demonstrated satisfactory internal consistency (*α* = 0.90 – 0.80), as well as good convergent validity with the Brief Symptom Inventory (BFI; 52) and the State-Trait Anxiety Inventory (STAI; 53), with correlations ranging from *r* = .53 and *r* = .71. Its brevity, strong psychometric properties, and ease of administration make it particularly suitable for use in clinical and community-based settings, including vulnerable populations such as those in assisted living facilities.

The scale consists of 10 items assessing symptoms of depression and anxiety over the past 30 days, rated on a 5-point scale. For the present study, the items were rephrased to assess symptoms over the past seven days only. The 5-point scale included the following response options: “None of the time” (1), “A little of the time” (2), “Some of the time” (3), “Most of the time” (4), and “All of the time” (5). Example items include: “During the last 7 days, about how often did you feel tired out for no good reason?” and “During the last 7 days, about how often did you feel restless or fidgety?” Participants’ responses were summed to create a total score ranging from 10 to 50 in total. Higher scores reflected greater emotional suffering. Psychological distress was assessed at baseline, each session, post-intervention, and follow-up.

### 2.7 Statistical analyses

All statistical analyses were conducted using the IBM SPSS Statistics version 30 software package.

#### 2.7.1 Data preprocessing

In accordance with the intent-to-treat (ITT) approach (54), all participants were retained in the analyses to ensure an unbiased estimation of treatment effect in a real-world context characterized by non-compliance and dropouts. Similarly, outliers were identified and reported via boxplots but retained in the analyses to reflect the natural heterogeneity of the sample and preserve data integrity.

Cases with missing values at baseline were excluded from further analyses. For cases where more than 50% of a scale was incomplete, missing values were imputed using the Last Observation Carried Forward (LOCF) method, whereby the participant’s last observed value was carried forward to replace subsequent missing entries. However, when more than 50% of a scale was completed, but isolated items were missing or erroneously marked, the mean value of the completed items was imputed for the missing responses, to allow for computation of total scores.

#### 2.7.2 Data analysis strategy

To test the hypotheses, six paired-samples *t*-tests were conducted to examine mean differences across outcome measures. In cases where the assumption of normality was violated, the Wilcoxon signed-rank test was used. To control for the increased risk of Type I error due to multiple comparisons, Bonferroni correction was applied separately within each outcome domain (baseline–post and post–follow-up comparisons for three outcomes; adjusted α = 0.05/3 = 0.017). Cohen’s d was reported as the effect size estimate and using standard benchmarks proposed by Cohen (55), where *d* ≈ 0.20 indicates a small effect, *d* ≈ 0.50 a medium effect, and *d* ≈ 0.80 a large effect. For paired-samples comparisons, *d* was calculated by dividing the mean difference between time points by the standard deviation of the difference scores (i.e., *d* = *M*_diff/_ *SD*_diff_), in line with recommendations for within-subject designs. One-tailed p-values were reported, reflecting our strictly unidirectional hypotheses of improvement (i.e., increases in goal attainment and self-efficacy, decreases in psychological distress). While one-tailed testing is less conservative and precludes detection of unexpected changes in the opposite direction, we considered this justified in a small-N pilot feasibility context with predefined directional hypotheses based on prior DBT-ST research.

Given the small sample and missing data across time points, we prespecified simple, contrast-focused within-subject comparisons as our primary analyses. This approach maximized transparency and interpretability for the two clinically most relevant transitions while avoiding additional assumptions (e.g., sphericity) and listwise deletion that are typical of repeated-measures ANOVA, both of which can substantially reduce power in already small samples. We acknowledge that a repeated-measures ANOVA or, preferably, a linear mixed-effects model (LMM) could provide a unified test of change over time and handle unbalanced data more efficiently. However, we chose the paired-comparison strategy as the primary analysis to align with the study’s feasibility focus and predefined clinical contrasts, and to maintain conservative Type I error control in a small-N, pilot context. The absence of a control group further limits causal inference irrespective of model choice; we therefore prioritized clarity of the targeted pre–post and post–follow-up contrasts. We note this as a methodological limitation and recommend LMMs as the primary analytic framework in larger, future confirmatory studies.

## 3 Results

**Table 1** presents the demographic characteristics of the DBT skill trainers, including their age, sex, the facility they are employed at, and their professional designation. There were twice as many female skill trainers as there were male. The Aktive Großfamilie facility had only one skill trainer available, who was supervised by a clinical health psychologist of the Heimo-Gastager-Haus.

**Table 1:** Demographic and professional characteristics of skills trainers.

Variable	N (%)	M (SD)
Age		44.92 (7.73)
Sex		
Male	4 (33.3)	
Female	8 (66.7)	
Facility		
Heimo Gastager Haus	3 (25.0)	
Südhof	5 (41.7)	
Tauernhof	3 (25.0)	
Aktive Großfamilie	1 (8.3)	
Professional Designation		
Psychologist	4 (33.3)	
Clinical and Health Psychologist	5 (41.7)	
Psychotherapist	1 (8.3)	
Social Education Worker	2 (16.7)	

N = 12. Percentages may not sum to 100% due to rounding.

Sample demographics of the patient sample are provided in **Table 2**. The sample consisted of 52 participants across all four facilities. Regarding sex distribution, the sample included more male residents (59.6%) than female residents (40.4%). Participants were nearly evenly distributed across three of the four facilities (Südhof and Tauernhof: n = 16 each; Aktive Großfamilie: n = 12), while the Heimo Gastager Haus contributed fewer (n = 8). Most participants were not currently undergoing psychotherapy (59.6%) and had previous experience with DBT skills (57.7%). Two participants failed to indicate whether their prior experience. The most prevalent diagnostic category (considering all diagnoses to account for comorbidities) was F20-29 (schizophrenia spectrum disorders), comprising 80.8% of the sample. Other diagnostic categories such as F30-39 (mood disorders) and F60-F69 (personality disorders) were represented to a lesser extent.

**Table 2:** Sample demographic characteristics and baseline measures.

Variable	N (%)	M (SD)
Age		45.73 (13.55)
Sex		
Male	31 (59.6)	
Female	21 (40.4)	
ICD-10 Diagnoses (including comorbidities)		
F10-F19	7 (13.5)	
F20-F29	42 (80.8)	
F30-F39	8 (15.4)	
F40-F49	9 (17.3)	
F60-F69	10 (19.2)	
F90-F99	1 (1.9)	

N = 52. Diagnostic categories F40-F59, F70-F79, and F80–89 were excluded due to lack of cases. ICD-10 diagnostic categories are not mutually exclusive due to comorbidity. Percentages may not sum to 100% due to rounding adjustments.

Attendance was complete, with all participants present at all eight DBT-ST sessions. In contrast, not all participants completed the self-report questionnaires at every time point. For goal attainment, completion rates were 77% at baseline, 94% at post-intervention, and 96% at follow-up. For self-efficacy, completion rates were 94% at baseline and 98% at both post-intervention and follow-up. For psychological distress, completion rates were 96% at baseline and 98% at both subsequent assessments.

Assumption checks for the paired-samples t-tests indicated violations of normality for the follow-up minus post-intervention difference scores in goal attainment, and self-efficacy, based on the Shapiro-Wilk test, skewness, kurtosis, and Q-Q plot inspection. For psychological distress, although the Shapiro-Wilk test was significant (*p* = .002), the kurtosis (1.66), skewness (-0.86), and Q-Q plot indicated a mild deviation. Given these circumstances, Wilcoxon signed-rank tests were performed for follow up vs. post-intervention comparisons to ensure robustness of the results. All other assumption checks were within accepted parameters.

Results of the paired-samples t-tests for baseline vs. post-intervention differences are shown in **Table 3**. Goal attainment and psychological distress showed significant changes at α = .05, but these did not remain significant after Bonferroni correction (adjusted α = .017). Effect sizes indicated small improvements, with goal attainment increasing by 11% (d = 0.28) and distress decreasing slightly (d = 0.25).

**Table 3:** Paired-samples t-test results from baseline (week 1) to post-intervention (week 8).

Outcome Measure	Week 1		Week 8		T(df)	P (one-tailed)	Cohen’s d
	M	SD	M	SD			
Goal Attainment	45%	30%	56%	30%	-1.80 (39)	.040	0.28
Self-Efficacy	3.39	0.85	3.50	0.83	-0.84 (48)	.202	0.12
Psychological Distress	23.75	6.98	22.00	7.50	1.87 (49)	.034	0.26

Each t-test compares Week 1 (baseline) to Week 8 (post-intervention) scores for goal attainment (n = 40), self-efficacy (n = 49), and psychological distress (n = 50). Two participants provided no usable baseline data, and one of these also missed post-intervention and follow-up. One participant did not complete the ASKU at baseline, and 10 others did not provide baseline goal attainment scores (with one also missing post-intervention and another missing both post- and follow-up). Cases with missing data were excluded only from analyses requiring the respective variable.

Wilcoxon signed-rank tests revealed no significant difference in goal attainment between post-intervention week 8 (*Mdn* = 50%, *IQR* = 45%) and follow-up week 10 (*Mdn* = 50%, *IQR* = 60%), *Z* = -.36, *p* = .359, *r* = .05; self-efficacy between post-intervention week 8 (*Mdn* = 0.33, *IQR* = 1.00) and follow-up week 10 (*Mdn* = 3.00, *IQR* = 1.00), *Z* = -.72, *p* = .237, *r* = .10; or psychological distress between post-intervention week 8 (*Mdn* = 22.00, *IQR* = 12.50) and follow-up week 10 (*Mdn* = 24.00, *IQR* = 12.50), *Z* = -.98, *p* = .165, *r* = .14.

## 4 Discussion

The present study examined the effects of an 8-week DBT skills training intervention on goal attainment, self-efficacy, and psychological distress as a marker for symptom severity among residents of assisted living facilities at post-intervention and two-week follow-up. The emphasis of this study lies less on efficacy and more on feasibility: the program was successfully implemented in four assisted living facilities, achieved full attendance, and was integrated into the existing daily structure. While the intervention led to some changes across time, not all results reached statistical significance. Specifically, goal attainment increased, and psychological distress decreased after the intervention compared to baseline, with small effect sizes. While the effects reached conventional significance levels (*p* <.05), they did not remain significant after Bonferroni correction for multiple comparisons. These findings appear sensitive to Type I error correction; while small, they may still hold clinical relevance in feasibility research but should be regarded as preliminary. No significant changes in the three outcome variables emerged between post-intervention and follow-up. This lack of further gains is not unexpected, as no intervention took place during the follow-up interval; in such chronic populations, maintaining initial improvements may be a more realistic goal than continued progress in the absence of booster sessions or ongoing support. Notably, self-efficacy, despite being a core target of DBT-ST, showed little change. This may reflect the limited intensity of an 8-week program in a long-term care context, or that some participants entered with moderate self-efficacy, leaving limited scope for measurable improvement.

While previous research on DBT-based interventions in clinical populations has reported moderate to large effect sizes, the present study found consistently small effects (d < 0.30). This discrepancy may be attributed to differences in sample characteristics, intervention setting, and intervention intensity. Unlike tightly controlled clinical trials, the current study was conducted in real-world assisted living settings. Participants had chronic psychiatric conditions, often including cognitive impairments and limited treatment engagement. These factors, together with the exclusive reliance on self-report measures, may have further constrained the detection of change, as impaired insight and concentration difficulties can reduce the accuracy and sensitivity of self-assessments. The issue is discussed further below. Furthermore, the adapted group-based format and the presence of concurrent interventions (e.g., individual therapy) may have reduced the specificity and magnitude of observed effects. Lastly, self-report measures may have been less sensitive to change in this population, further contributing to the small observed effect sizes. However, even in structured inpatient settings, effect sizes are not necessarily large, as the study by Probst et al. (56) demonstrated. They reported a small-to-moderate effect size (d = 0.38) for symptom reduction on the Borderline Symptom List-23 (BSL-23) in an intention-to-treat analysis of a 5-week inpatient DBT program. The similarly small effect size found in the current study for psychological distress suggests a comparable magnitude of change, albeit in a substantially different context. As noted, the study setting, heterogeneity of the sample, and the lower treatment intensity may play an important role. Probst et al. (56) also argue that the treatment length and different self-reported outcome measures may influence such benchmark comparisons. Although statistical significance was not maintained in the present study, the comparable effect sizes may suggest that even low-intensity, community-based DBT Skills Training holds potential for symptom relief in highly vulnerable populations. However, the absolute changes were small (e.g., distress scores declined from 23.75 to 22.0; goal attainment rose from 45% to 56%), and it remains uncertain whether such shifts represent clinically meaningful improvements in daily functioning. At the same time, feasibility indicators such as full attendance and smooth integration into facility routines underscore the practicality of DBT-ST in long-term care environments. These findings should be interpreted cautiously and viewed as preliminary, warranting replication in larger, fully powered studies.

The discussed findings also carry concrete implications for practice. Successful implementation requires adequate staff training, as staff members play a central role in encouraging skill use outside the group sessions and supporting participants with reminders and prompts. Materials and exercises should be adapted to accommodate cognitive impairments common in this population, for example by simplifying worksheets, using visual aids, and allowing more repetition. Embedding DBT-ST within the routine care structure, such as offering sessions in place of existing group therapy or house meetings, appears feasible and may facilitate participation without overburdening residents. Taken together, these considerations suggest that DBT-ST can be incorporated into assisted living facilities with relatively low structural changes, provided that training and support for staff and adaptations for cognitive limitations are ensured.

### 4.1 Limitations

The current study may have lacked sufficient power to detect small but potentially meaningful effects, particularly given the limited variability introduced by diagnostic homogeneity. The use of a strict Bonferroni correction, while cautious, may have further increased the risk of Type II error in this small-N context. Future studies might consider less conservative adjustments, such as the Holm–Bonferroni method, to balance Type I and Type II error risks more appropriately. The sample was recruited on a convenience basis and was predominantly composed of individuals with schizophrenia spectrum disorders (80.8%). This reduces representativeness and limits generalizability to broader transdiagnostic populations. As such, while the intervention was offered to residents with diverse psychiatric diagnoses, the conclusions regarding transdiagnostic effects must be interpreted with caution. Moreover, the assisted living residences included in this study resemble psychiatric long-term care settings, which further limits the generalizability of the findings. Results may therefore not extend to other populations or contexts, such as general elderly assisted living facilities or outpatient care. This suggests that either the true effects were smaller than anticipated, or that other methodological factors, such as measurement limitations and restricted variance, may have obscured real effects. In addition, the modest sample size and missing data across time points further limited statistical power. With effect sizes around d = 0.25, a substantially larger sample would be needed to achieve significance, and the possibility of false-negative findings, particularly regarding self-efficacy, cannot be excluded. In future studies, a formal power analysis (e.g., using G*Power) should be conducted to plan appropriate sample sizes. Strategies to reduce missing data, such as ongoing engagement efforts and collecting follow-up assessments even from participants who miss post-assessment, would also be beneficial. Although we applied an ITT approach with last-observation-carried-forward (LOCF) for within-program missing sessions, missing entire time-points reduces power and may bias results if dropouts differ from completers. Notably, the reliance on self-report measures in a sample largely composed of individuals with schizophrenia spectrum diagnoses may have introduced measurement error. Schizophrenia often entails impaired insight, cognitive distortions, and reduced emotional awareness. Lysaker and Dimaggio (57) found that individuals with schizophrenia often struggle with accurate self-appraisal, particularly regarding functioning and emotional experience. Similarly, Lincoln et al. (58) demonstrated that self-reports in schizophrenia may be distorted by delusions, poor insight, and cognitive impairments. Feedback from skills trainers in this study reflected these challenges: participants often had difficulties concentrating in the group context, completing questionnaires without close support, or understanding the language of the training material. As the outcomes of the current study rely on accurate introspection, their reliability in this sample is likely compromised, underscoring the need for complementary assessment methods in such settings. To address this in future research, a more heterogeneous, transdiagnostic sample should be recruited to ensure broader generalizability and variability. Additionally, and particularly in the context of assisted living facilities, which predominantly house individuals with schizophrenia, intervention materials should be further simplified, for instance by incorporating visual cues, audiobook formats or video-based instructions and examples. Alternative assessment methods should also be considered, such as observer- or clinician-rated scales, semi-structured interviews, or behavioral indicators. Ecological momentary assessments (EMAs) may help reduce retrospective cognitive bias. Additionally, metacognitive measures, such as the abbreviated version of the Metacognition Assessment Scale (MAS-A; 57, should be employed to evaluate the realism of self-assessments.

Furthermore, the potential influence of psychopharmacological medication must also be considered, as many antipsychotic and other psychotropic agents are known to affect cognitive functioning, emotional processing, and interoceptive accuracy. For example, antipsychotic medications can induce sedation, dampen affective responsiveness, and impair attention or working memory (59), all of which are critical for accurate self-reflection and reliable questionnaire completion. In addition to cognitive and emotional effects, certain medications may directly interfere with the perception and intensity of bodily sensations, a central component of many DBT skills. For instance, skills involving sensory grounding techniques (e.g., applying spiky massage balls to the skin, eating spicy foods like chili, or using strong scents such as peppermint or lavender) rely on an individual’s intact capacity to register and differentiate physical sensations. However, psychotropic agents, particularly antipsychotics, have been shown to blunt interoceptive awareness and reduce somatosensory sensitivity (60). This represents a major limitation, as medication effects may undermine the validity of self-reported outcomes by reducing concentration, affective responsiveness, and insight. Simultaneously, they may attenuate the actual experiential effectiveness of core DBT skills, such as sensory grounding and mindfulness practices. These influences may therefore affect both the measured and true effectiveness of the intervention, particularly in populations with severe mental illness. Future research should systematically assess medication type, dosage, and side-effect profiles, and consider them as potential covariates or moderators in intervention studies.

In addition, the multi-site implementation introduces potential confounds. Trainer qualifications ranged from fully licensed psychologists and psychotherapists to social educational workers in supervision, which may have affected delivery fidelity and participant outcomes. Facility contexts also differed: one residence functioned as a transitional facility, where care structures and patient profiles may have differed from long-term homes, with participants possibly less severely ill. Although participant groups remained stable during the intervention, such contextual variability could have influenced engagement, support, and treatment responsiveness independent of DBT-ST. As participants were not randomized by site, it is not possible to disentangle intervention effects from these site-specific influences, which should be considered in the interpretation of results.

Finally, it is important to acknowledge that the intervention was implemented within a naturalistic, real-world setting, in which participants continued to receive other therapeutic services alongside DBT-ST. These included individual psychotherapy, ergotherapy (occupational therapy), and additional group-based interventions such as psychoeducation or social skills training. The simultaneous administration of multiple therapeutic programs introduces potential confounds, as improvements in outcomes may not be attributable solely to DBT-ST. Conversely, overlapping content or therapeutic fatigue could have diluted its specific effects. Future studies should attempt to systematically document concurrent treatments and, where possible, control for their influence through study design or statistical adjustment.

Another limitation is the potential influence of site and trainer variability. The participating facilities differed in structure (e.g., one transitional with higher turnover, others long-term residences), and trainers varied in level of experience, ranging from qualified staff to trainees under supervision. As the sample was not randomized by site, contextual differences such as facility culture, patient characteristics, or trainer expertise could have influenced outcomes. In addition, nearly 60% of participants had prior exposure to DBT skills, which may have reduced room for measurable improvement in some individuals or, conversely, facilitated skill uptake in others. With the current sample size, such potential moderating effects could not be examined, but they warrant systematic consideration in future research.

In addition, our analytic strategy, while transparent and aligned with predefined contrasts, is suboptimal for longitudinal data. More powerful approaches, such as repeated-measures ANOVA or linear mixed-effects models (LMMs), can jointly model all time points and better accommodate missing data. Relying on multiple paired t-tests may have reduced statistical power and precision in this small treatment group. Furthermore, the absence of a control group represents a major limitation, as improvements cannot be attributed solely to DBT-ST in the presence of concurrent interventions and natural fluctuations. Future research should therefore employ LMMs within larger samples and include appropriate control groups to strengthen causal inference and provide a more rigorous test of intervention effects. Although supervision was provided through team meetings, the absence of structured fidelity monitoring (e.g., adherence checklists or independent ratings) represents a limitation, as intervention integrity could not be systematically evaluated. Finally, the use of one-tailed tests may be viewed as less conservative, as it reduces sensitivity to unexpected changes in the opposite direction. While this choice was aligned with our *a priori* hypotheses of improvement, future studies should employ two-tailed testing to allow for more balanced inference.

Attendance was complete, indicating that integration of DBT-ST into daily structures is feasible. However, completion of self-report questionnaires was somewhat lower, particularly for the goal attainment measure at baseline. As noted previously, the reliance on self-report may be problematic in this population, and the current findings reinforce the need for complementary observer- or clinician-rated assessments in future studies to ensure more complete and valid outcome data.

Despite these limitations, this study provides preliminary insights into the feasibility and challenges of implementing DBT-ST in assisted living facilities. While DBT based interventions have been applied in various settings, including inpatient and outpatient units, residential programs for adolescents, and community mental health centers, there is little evidence of its application specifically within assisted living facilities. This study may therefore be among the first to explore the feasibility of DBT-ST in this context. It offers a novel contribution to the field by providing a foundation for further investigation into context-sensitive adaptations of DBT in long-term care environments. Attendance and integration into daily routines suggest that the program is acceptable in this context, but observed effects were small and did not withstand correction for multiple comparisons. These findings should therefore be regarded as tentative. Replication in larger, more heterogeneous samples, the inclusion of control groups, longer-term follow-up assessments, and the use of more robust, multimodal measurement approaches will be essential to draw firmer conclusions about the effectiveness and applicability of DBT-ST. In addition, delivery methods, assessment tools, and population targeting, as outlined above, should be refined to optimize the effectiveness and real-world utility of DBT-based interventions in complex long-term care environments.

## Data Availability

The raw data supporting the conclusions of this article will be made available by the authors, without undue reservation.
